# Severe Urine Retention and Deep Vein Thrombosis: A Case Report and Review of the Literature on an Unusual Association

**DOI:** 10.7759/cureus.37568

**Published:** 2023-04-14

**Authors:** Elrazi A Ali, Colleen Achong, Nabin KC, Danilo Enriquez, Kalpana Panigrahi, Abdalla Fadul, Eihab A Subahi, Ahmed Abdallah

**Affiliations:** 1 Internal Medicine, One Brooklyn Health - Interfaith Medical Center, Brooklyn, USA; 2 Pulmonology, One Brooklyn Health - Interfaith Medical Center, Brooklyn, USA; 3 Internal Medicine, Hamad Medical Corporation, Doha, QAT; 4 General Internal Medicine Fellowship Program, Medical Education, Hamad Medical Corporation, Doha, QAT; 5 Nephrology, University of Arkansas for Medical Sciences, Little Rock, USA

**Keywords:** young adult, bladder outlet obstruction, deep vein thrombosis (dvt), thrombosis, urine retention

## Abstract

Acute urine retention is the most common urologic emergency, and it usually presents with abdominal pain and an inability to pass urine. The distended bladder in urine retention can be enormously large, raising the intra-abdominal pressure and compressing the iliac veins draining the lower limbs and pelvis. Many cases have been reported to have deep vein thrombosis (DVT)-like features with urine retention that resolves with bladder decompression. In rare cases, urine retention can lead to DVT, particularly in young patients. We report a case of a young female patient with a huge distended bladder who devolved extensive venous thrombosis bilaterally. The report sheds light on this unusual complication of acute urine retention and reviews the existing literature on the topic.

## Introduction

Venous thromboembolism (VTE) is a common presentation and complication associated with various medical conditions. It is classified as provoked or unprovoked based on the presence of precipitating factors of the thrombotic condition [1]. The primary mechanism by which thrombosis occurs is Virchow’s triad of venous stasis, hypercoagulable conditions like pregnancy or inherited thrombophilia, or endothelial injury, as in patients in the postoperative state [2]. Among the unusual causes of thrombosis and venous stasis is organomegaly, causing compression of the veins draining the lower limbs and pelvis, and reducing the venous return. In rare cases, urine retention can cause a significant rise in intra-abdominal pressure. The urinary bladder can expand and have the capacity to accommodate several liters of urine. Some reports have shown that the bladder could accommodate several liters of urine. This case describes a case of urine retention and a huge urinary bladder, reaching a size of 9 liters, the largest bladder size ever reported in a female patient; we also engage in a review of previous similar cases of an unusual cause of VTE.

## Case presentation

A 37-year-old woman with a past medical history of bipolar disorder was admitted to the hospital with a complaint of abdominal pain and subsequently diagnosed with acute pancreatitis secondary to gallbladder stones (biliary pancreatitis). After the resolution of pancreatitis on day five of admission, she had a laparoscopic cholecystectomy and was discharged home. Three days after discharge, she had nausea, vomiting, and abdominal pain associated with abdominal distension. The pain and nausea worsened, and after two weeks, she presented to the emergency department (ED) as the pain was not tolerable. On physical exam, she had a distended abdomen and bilateral lower limb edema more prominent on the left side.

**Figure 1:**
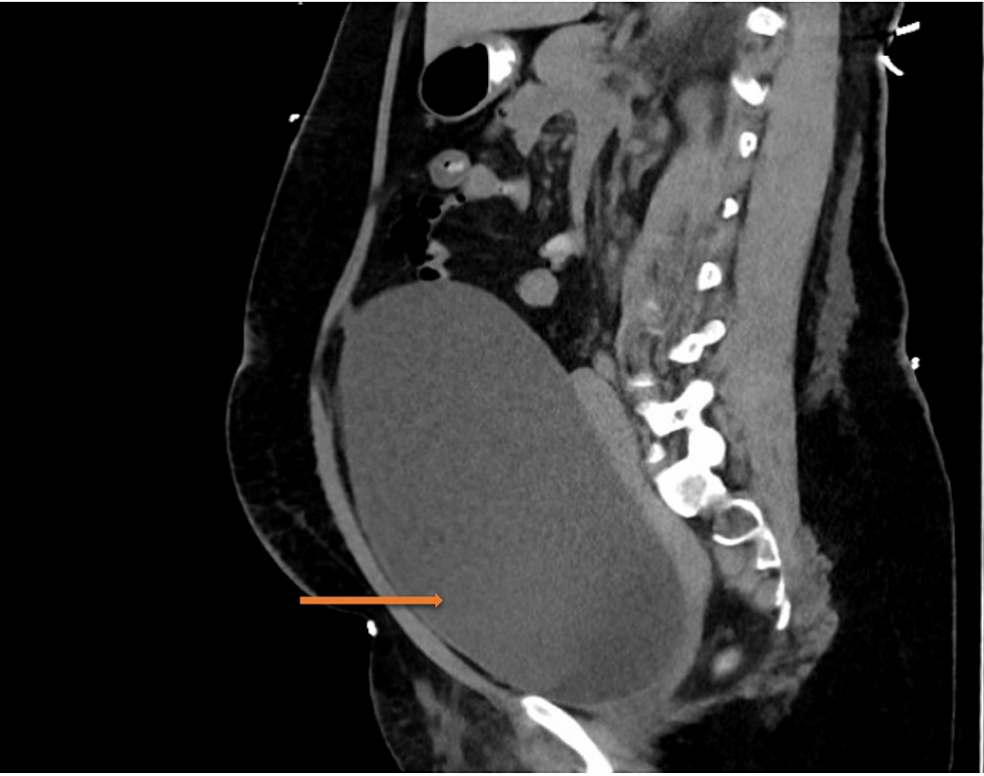
CT scan of the abdomen showing a huge distended bladder (arrow) CT: computed tomography

Initial blood investigation revealed acute kidney injury (AKI) with hyperkalemia without ECG changes; other blood investigations are shown in Table 1. Lower limb Doppler showed bilateral femoral vein thrombosis. In the ED, she had an abdominal CT scan, which showed a huge distended bladder (Figure 1) with reported mild bilateral hydronephrosis with bilateral hydroureter.

**Table 1:** Initial blood investigation on admission

Variables	Result	Reference range
White blood cell count	6.9	4.5-11 × 10^9^/L
Hemoglobin	10.9	11-15 g/dl
Platelet count	232	130-400 × 10^9^/L
Blood urea nitrogen	101	7-18.7 mg/dl
Creatinine	15.7	0.57-1.11 mg/dl
Sodium	138	136-145 mmol/L
Potassium	6.3	3.5-5.1 mmol/L
Aspartate transaminase	20	5-34 U/L
Alanine transaminase	81	10-55 U/L
Albumin	3.8	3.5-5.2 g/dl

Foley's catheter was inserted, and 9 liters of urine was drained over 18 hours. Foley's catheter was clamped after draining 4 liters to avoid rapid decompression of the distended urinary tracts. During the following 24 hours, the patient was still polyuric, and she received IV fluid. She was started on heparin infusion for VTE treatment. After 48 hours of decompression, the renal function normalized, and the patient was switched to a therapeutic dose of enoxaparin. On day five of admission, she was switched to rivaroxaban, and two days later, she was discharged home with advice to follow up. Thrombophilia screening was sent and came back negative for antiphospholipid antibodies protein C and protein S deficiency. The patient was labeled as a case of provoked thrombosis related to urine retention due to the postoperative condition and anticholinergic effects of antipsychotics.

## Discussion

VTE is one of the major causes of morbidity and mortality worldwide [3]. Thrombosis is a complication that can be difficult to predict. Common causes of deep vein thrombosis (DVT) include a hypercoagulable state, like pregnancy, following trauma and fracture, orthopedic surgery, active malignancy, and inherited thrombophilias. Thrombosis in young patients can be seen with less common causes like infection with severe acute respiratory syndrome coronavirus 2 (SARS-CoV-2), immune thrombocytopenic purpura (ITP) [4], May-Thurner syndrome, and celiac disease [5]. Therefore, prophylaxis against VTE is one of the major ways of reducing morbidity and mortality in hospitalized patients.

Acute urine retention usually presents with a painful inability to void. The management of urine retention includes bladder decompression by catheterization, either by urethral or suprapubic catheter, based on the patient's condition. Several cases in the literature have shown that bladder distention may cause venous compression or obstruction leading to lower limb edema without thrombosis [6,7], and in severe cases, it may even cause transient inferior vena cava obstruction [8]. The increase in bladder distention is associated with increased pressure in the iliac veins [9]. However, intravascular thrombosis due to an enlarged bladder is very rare.

Our patient had a past medical history of bipolar disorder; she had received treatments that can cause urine retention. Furthermore, she had pancreatitis and undergone cholecystectomy, and experienced prolonged immobility that may increase the risk of both urine retention and DVT. Additionally, the prolonged period of obstruction of two weeks' duration and the delayed presentation of the patient likely resulted in prolonged venous stasis and, subsequently, the development of thrombosis [2].

The most common cause of urine retention in adults is benign prostatic hyperplasia in male patients [10,11]. Other causes of urine retention include urolithiasis, strictures, and neurogenic bladder or inefficient detrusor muscle [12]. Additionally, medication with anticholinergic effects or with β3 adrenergic agonist activity can precipitate urine retention. In females, large pelvic masses are associated with an increased risk of DVT. This has been reported even with benign tumors like urine myoma, by causing compression of the pelvic veins and subsequent venous stasis and clot formation. A similar mechanism is seen in May-Thurner syndrome, in which the iliac veins are compressed by the nearby artery, leading to venous obstruction and DVT. To our knowledge, this is the first case of urine retention in a female patient that is associated with venous thrombosis.

A review of the literature revealed a few cases (five) of thrombosis following urine retention. The first reported case of VTE related to bladder distention was described in a pediatric patient in 1960 [13]. The patient was a three-week-old child with a urethral valve. All other patients were males above the age of 68 years (Table 2).

**Table 2:** Previous studies involving thrombosis secondary to urine retention N/A: not applicable

Study	Publication year	Patient age and gender	Presentation	Physical exam	Bladder size	Location	Urinary tract dilatation	Diagnostic method	Presence of pulmonary embolism	Cause of urine retention	Thrombophilia/risk factors of thrombosis	Treatment	Outcome/complication
Carlsson and Garsten [13]	1960	3 weeks, male	Abdominal mass; left leg becoming cold, pale, and cyanotic	A hard lobulated mass in the lower abdomen	120 ml	Left external and common iliac veins	Dilatation of renal pelvis and ureter	N/A	No	Urethral valve	No	Transurethral electroresection of the bladder neck and urethral valves	2 weeks after the intervention, the circulation in the left lower extremity was normal
Kawada et al. [14]	2018	75 years, male	Lower extremity weakness	Bilateral leg edema	N/A	Bilateral intrapelvic veins	N/A	Contrast-enhanced CT	Right pulmonary artery	Anticholinergic agent, propiverine, detrusor insufficiency	No	Rivaroxaban 30 mg	3 months after treatment, the patient reported no other events since the beginning of the treatment
Evans et al. [15]	1995	73 years, male	4 days of left lower extremity swelling	Left lower extremity pitting edema from the foot to the groin	Above the umbilicus to the epigastrium	Iliofemoral deep vein	Mild hydronephrosis	Doppler lower limb	No	BPH atonic bladder	No	N/A	After catheter insertion, the edema resolved within several hours
Umemura et al. [16]	2011	76 years, male	4 days of lower abdominal distention	Rigid abdomen	N/A	Right femoral vein	N/A	N/A	Yes	BPH atonic bladder	Chronic AF without anticoagulation therapy	Anticoagulation and catheterization	The patient became symptom-free and was discharged
Sharma et al. [17]	2014	68 years, male	Dyspnea	Extensive varicose veins bilaterally	5 liters	Bilateral large pulmonary emboli	N/A	CT	Yes	Unclear	No	NA	Required intermittent catheterization

All patients were males and older than 65 years, except one patient. All patients had lower limb clots; three patients had a pulmonary embolism in addition to lower limb thrombosis. All patients were screened for thrombophilia, and the workup was negative. The thrombus was seen in the right venous system, left venous system, and bilaterally. Two patients had hydronephrosis, and the other cases did not mention hydronephrosis.

## Conclusions

Urine retention should be recognized early and treated promptly as delayed treatment can precipitate venous obstruction and venous thrombosis, particularly in patients with an increased risk of thrombosis. Bladder distention causing thrombosis can occur, and it has been reported in pediatric patients as well as older adults and young females. Although urine retention is unusual in young females, early recognition is vital to prevent severe complications like thrombosis and pulmonary embolism.
